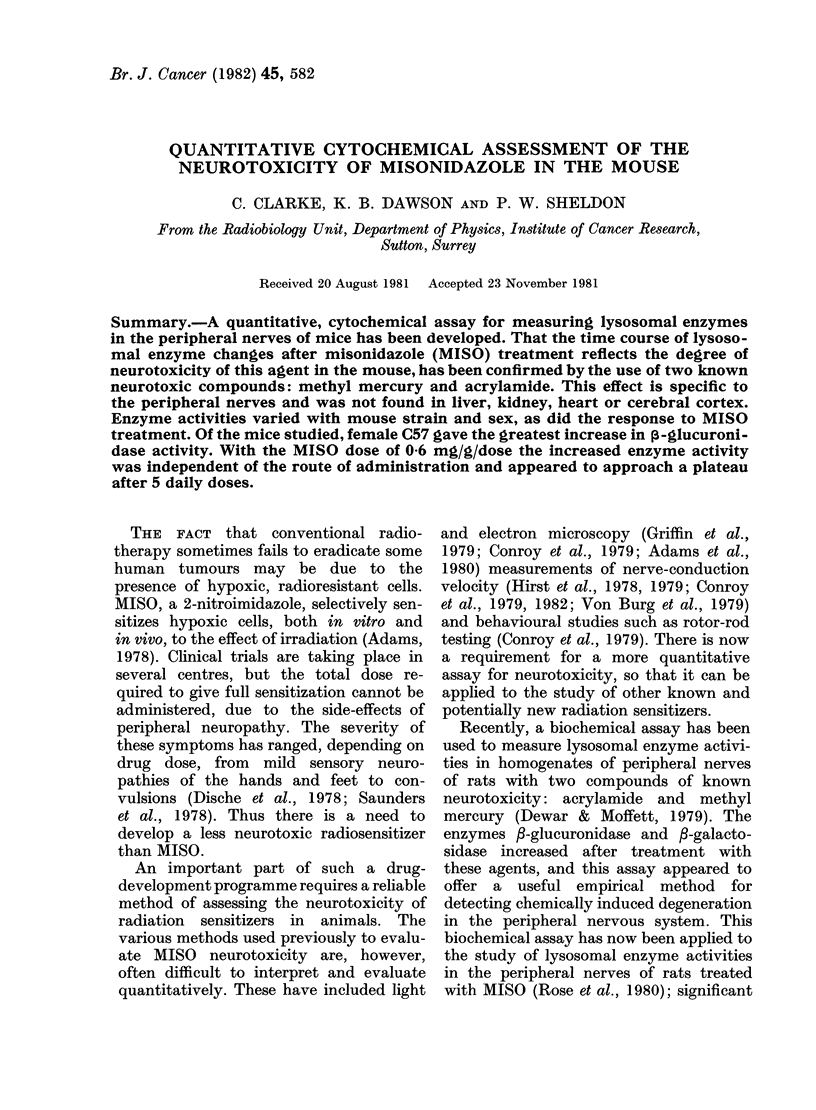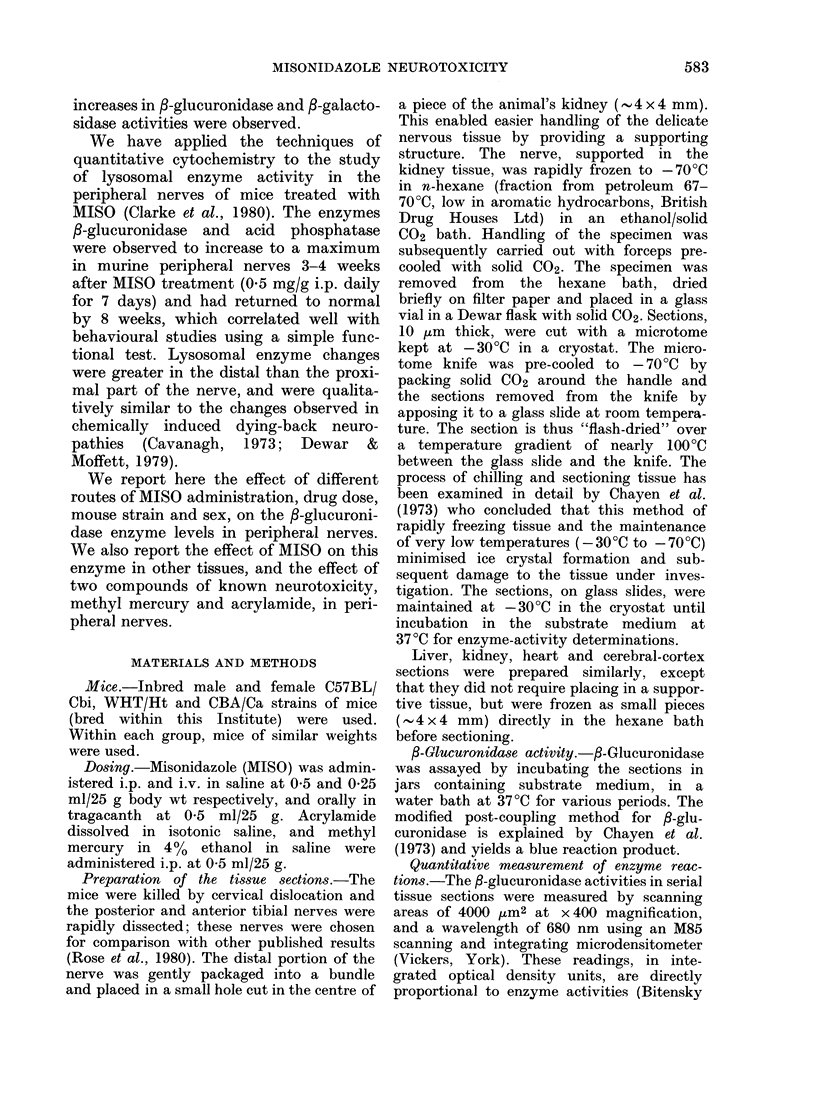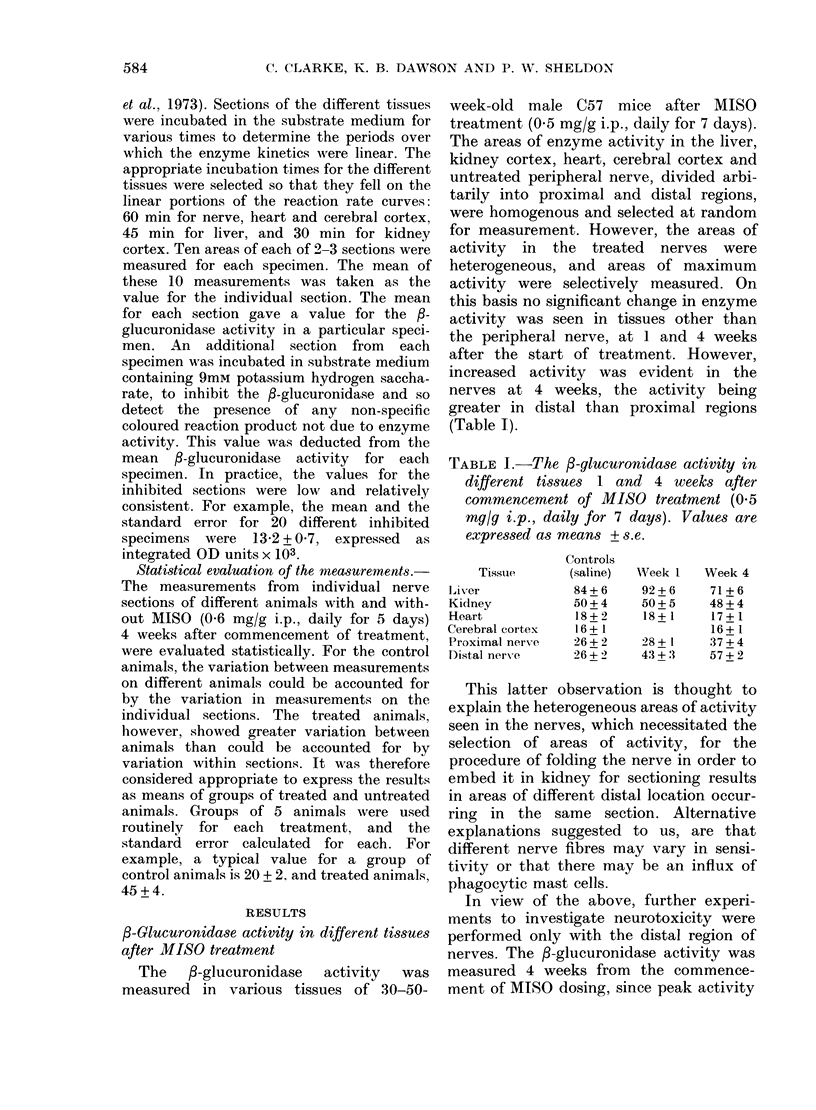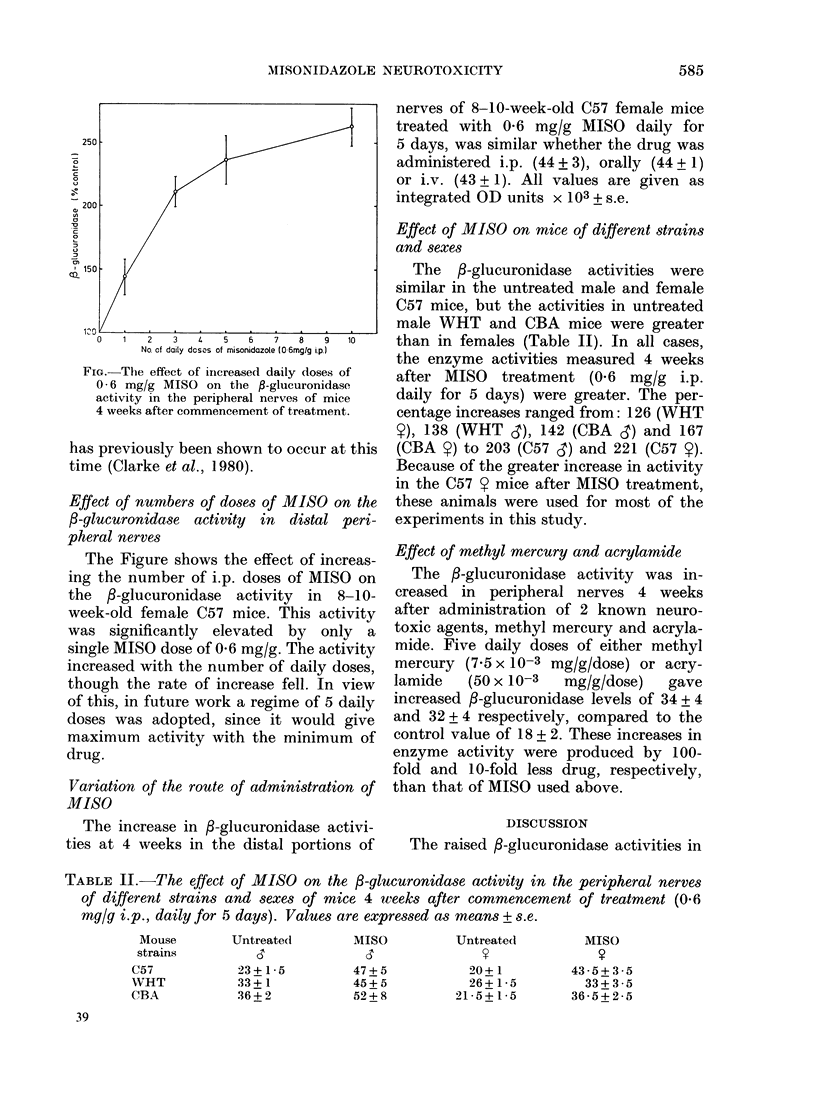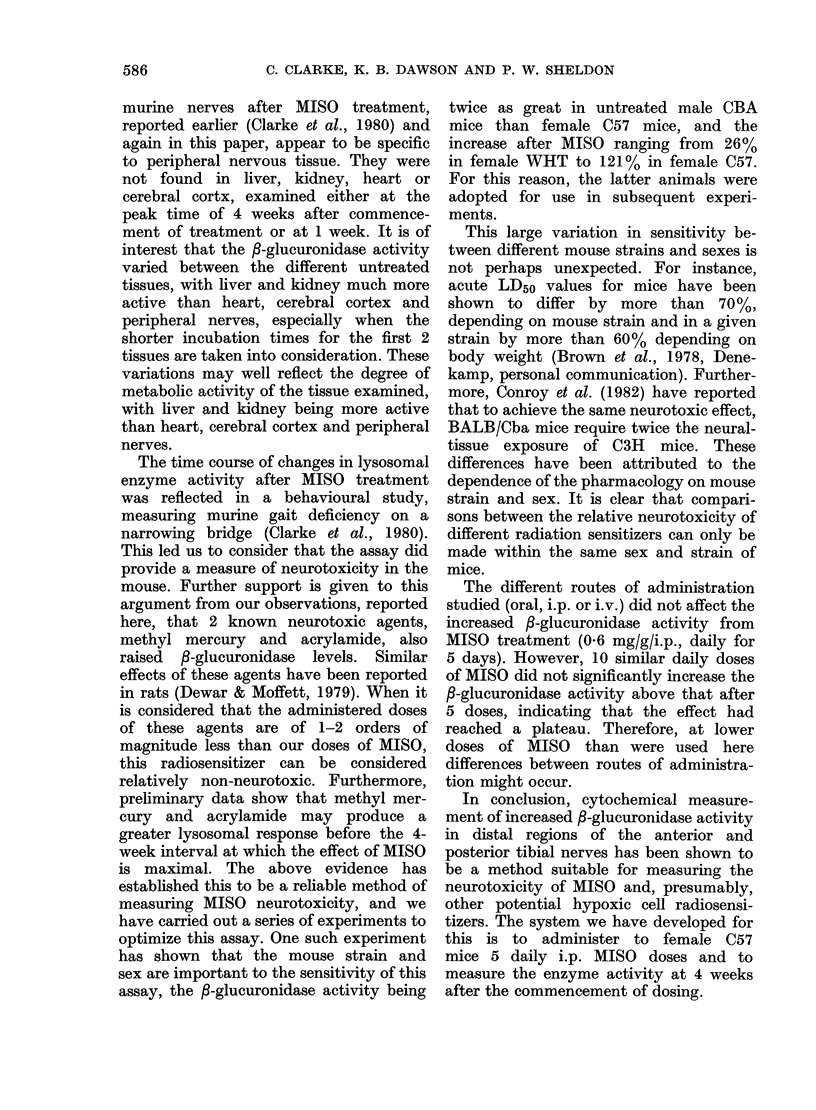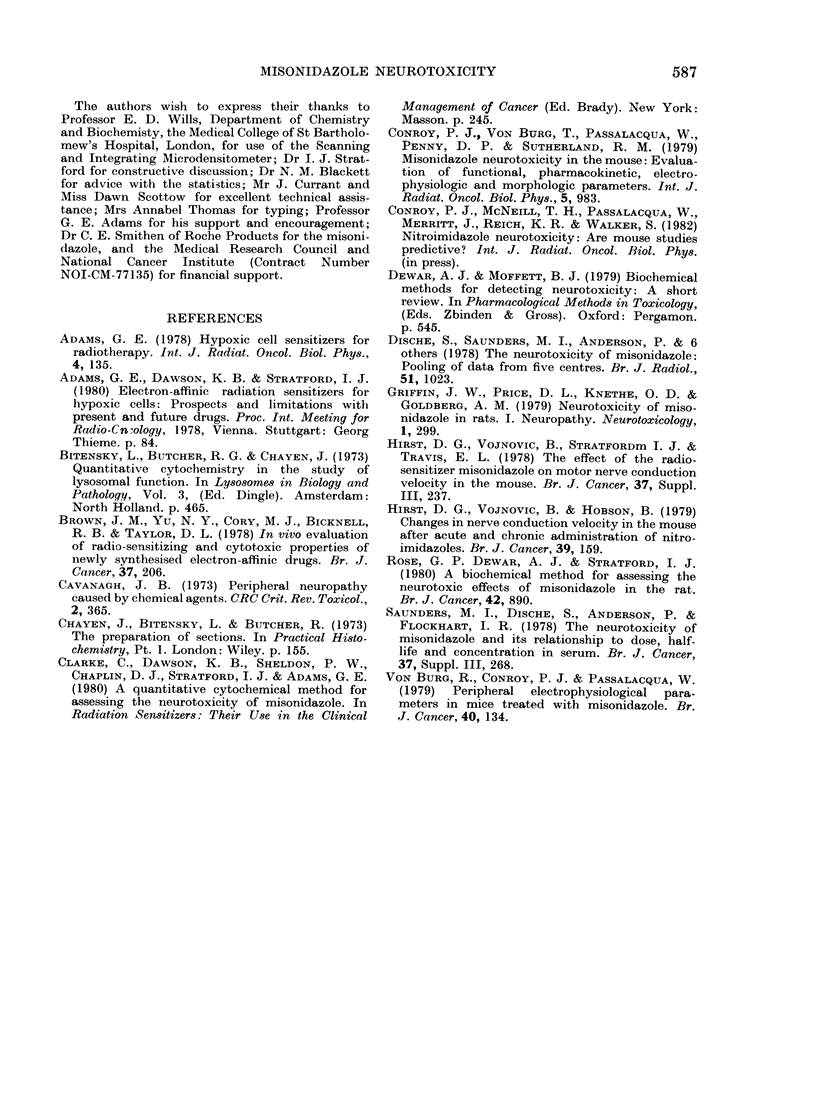# Quantitative cytochemical assessment of the neurotoxicity of misonidazole in the mouse.

**DOI:** 10.1038/bjc.1982.95

**Published:** 1982-04

**Authors:** C. Clarke, K. B. Dawson, P. W. Sheldon

## Abstract

A quantitative, cytochemical assay for measuring lysosomal enzymes in the peripheral nerves of mice has been developed. That the time course of lysosomal enzyme changes after misonidazole (MISO) treatment reflects the degree of neurotoxicity of this agent in the mouse, has been confirmed by the use of two known neurotoxic compounds: methyl mercury and acrylamide. This effect is specific to the peripheral nerves and was not found in liver, kidney, heart or cerebral cortex. Enzyme activities varied with mouse strain and sex, as did the response to MISO treatment. Of the mice studied, female C57 gave the greatest increase in beta-glucuronidase activity. With the MISO dose of 0.6 mg/g/dose the increased enzyme activity was independent of the route of administration and appeared to approach a plateau after 5 daily doses.


*Br. J. Cancer* (1982) **45**, 582

### QUANTITATIVE CYTOCHEMICAL ASSESSMENT OF THE NEUROTOXICITY OF MISONIDAZOLE IN THE MOUSE

C. CLARKE, K. B. DAWSON AND P. W. SHELDON

*From the Radiobiology Unit, Department of Physics, Institute of Cancer Research, Sutton, Surrey*

Received 20 August 1981 Accepted 23 November 1981

**Summary.—A quantitative, cytochemical assay for measuring lysosomal enzymes in the peripheral nerves of mice has been developed. That the time course of lysosomal enzyme changes after misonidazole (MISO) treatment reflects the degree of neurotoxicity of this agent in the mouse, has been confirmed by the use of two known neurotoxic compounds: methyl mercury and acrylamide. This effect is specific to the peripheral nerves and was not found in liver, kidney, heart or cerebral cortex. Enzyme activities varied with mouse strain and sex, as did the response to MISO treatment. Of the mice studied, female C57 gave the greatest increase in β-glucuronidase activity. With the MISO dose of 0·6 mg/g/dose the increased enzyme activity was independent of the route of administration and appeared to approach a plateau after 5 daily doses.**

THE FACT that conventional radiotherapy sometimes fails to eradicate some human tumours may be due to the presence of hypoxic, radioresistant cells. MISO, a 2-nitroimidazole, selectively sensitizes hypoxic cells, both *in vitro* and *in vivo*, to the effect of irradiation (Adams, 1978). Clinical trials are taking place in several centres, but the total dose required to give full sensitization cannot be administered, due to the side-effects of peripheral neuropathy. The severity of these symptoms has ranged, depending on drug dose, from mild sensory neuropathies of the hands and feet to convulsions (Dische *et al.*, 1978; Saunders *et al.*, 1978). Thus there is a need to develop a less neurotoxic radiosensitizer than MISO.

An important part of such a drug-development programme requires a reliable method of assessing the neurotoxicity of radiation sensitizers in animals. The various methods used previously to evaluate MISO neurotoxicity are, however, often difficult to interpret and evaluate quantitatively. These have included light and electron microscopy (Griffin *et al.*, 1979; Conroy *et al.*, 1979; Adams *et al.*, 1980) measurements of nerve-conduction velocity (Hirst *et al.*, 1978, 1979; Conroy *et al.*, 1979, 1982; Von Burg *et al.*, 1979) and behavioural studies such as rotor-rod testing (Conroy *et al.*, 1979). There is now a requirement for a more quantitative assay for neurotoxicity, so that it can be applied to the study of other known and potentially new radiation sensitizers.

Recently, a biochemical assay has been used to measure lysosomal enzyme activities in homogenates of peripheral nerves of rats with two compounds of known neurotoxicity: acrylamide and methyl mercury (Dewar & Moffett, 1979). The enzymes β-glucuronidase and β-galactosidase increased after treatment with these agents, and this assay appeared to offer a useful empirical method for detecting chemically induced degeneration in the peripheral nervous system. This biochemical assay has now been applied to the study of lysosomal enzyme activities in the peripheral nerves of rats treated with MISO (Rose *et al.*, 1980); significant increases in β-glucuronidase and β-galactosidase activities were observed.

We have applied the techniques of quantitative cytochemistry to the study of lysosomal enzyme activity in the peripheral nerves of mice treated with MISO (Clarke *et al.*, 1980). The enzymes β-glucuronidase and acid phosphatase were observed to increase to a maximum in murine peripheral nerves 3–4 weeks after MISO treatment (0·5 mg/g i.p. daily for 7 days) and had returned to normal by 8 weeks, which correlated well with behavioural studies using a simple functional test. Lysosomal enzyme changes were greater in the distal than the proximal part of the nerve, and were qualitatively similar to the changes observed in chemically induced dying-back neuropathies (Cavanagh, 1973; Dewar & Moffett, 1979).

We report here the effect of different routes of MISO administration, drug dose, mouse strain and sex, on the β-glucuronidase enzyme levels in peripheral nerves. We also report the effect of MISO on this enzyme in other tissues, and the effect of two compounds of known neurotoxicity, methyl mercury and acrylamide, in peripheral nerves.

### MATERIALS AND METHODS

*Mice.*—Inbred male and female C57BL/Cbi, WHT/Ht and CBA/Ca strains of mice (bred within this Institute) were used. Within each group, mice of similar weights were used.

*Dosing.*—Misonidazole (MISO) was administered i.p. and i.v. in saline at 0·5 and 0·25 ml/25 g body wt respectively, and orally in tragacanth at 0·5 ml/25 g. Acrylamide dissolved in isotonic saline, and methyl mercury in 4% ethanol in saline were administered i.p. at 0·5 ml/25 g.

*Preparation of the tissue sections.*—The mice were killed by cervical dislocation and the posterior and anterior tibial nerves were rapidly dissected; these nerves were chosen for comparison with other published results (Rose *et al.*, 1980). The distal portion of the nerve was gently packaged into a bundle and placed in a small hole cut in the centre of a piece of the animal's kidney (~4×4 mm). This enabled easier handling of the delicate nervous tissue by providing a supporting structure. The nerve, supported in the kidney tissue, was rapidly frozen to −70°C in *n*-hexane (fraction from petroleum 67–70°C, low in aromatic hydrocarbons, British Drug Houses Ltd) in an ethanol/solid $CO_2$ bath. Handling of the specimen was subsequently carried out with forceps pre-cooled with solid $CO_2$. The specimen was removed from the hexane bath, dried briefly on filter paper and placed in a glass vial in a Dewar flask with solid $CO_2$. Sections, 10 μm thick, were cut with a microtome kept at −30°C in a cryostat. The microtome knife was pre-cooled to −70°C by packing solid $CO_2$ around the handle and the sections removed from the knife by apposing it to a glass slide at room temperature. The section is thus "flash-dried" over a temperature gradient of nearly 100°C between the glass slide and the knife. The process of chilling and sectioning tissue has been examined in detail by Chayen *et al.* (1973) who concluded that this method of rapidly freezing tissue and the maintenance of very low temperatures (−30°C to −70°C) minimised ice crystal formation and subsequent damage to the tissue under investigation. The sections, on glass slides, were maintained at −30°C in the cryostat until incubation in the substrate medium at 37°C for enzyme-activity determinations.

Liver, kidney, heart and cerebral-cortex sections were prepared similarly, except that they did not require placing in a supportive tissue, but were frozen as small pieces (~4×4 mm) directly in the hexane bath before sectioning.

*β-Glucuronidase activity.*—β-Glucuronidase was assayed by incubating the sections in jars containing substrate medium, in a water bath at 37°C for various periods. The modified post-coupling method for β-glucuronidase is explained by Chayen *et al.* (1973) and yields a blue reaction product.

*Quantitative measurement of enzyme reactions.*—The β-glucuronidase activities in serial tissue sections were measured by scanning areas of 4000 μm² at ×400 magnification, and a wavelength of 680 nm using an M85 scanning and integrating microdensitometer (Vickers, York). These readings, in integrated optical density units, are directly proportional to enzyme activities (Bitensky

*et al.*, 1973). Sections of the different tissues were incubated in the substrate medium for various times to determine the periods over which the enzyme kinetics were linear. The appropriate incubation times for the different tissues were selected so that they fell on the linear portions of the reaction rate curves: 60 min for nerve, heart and cerebral cortex, 45 min for liver, and 30 min for kidney cortex. Ten areas of each of 2–3 sections were measured for each specimen. The mean of these 10 measurements was taken as the value for the individual section. The mean for each section gave a value for the β-glucuronidase activity in a particular specimen. An additional section from each specimen was incubated in substrate medium containing 9mM potassium hydrogen saccharate, to inhibit the β-glucuronidase and so detect the presence of any non-specific coloured reaction product not due to enzyme activity. This value was deducted from the mean β-glucuronidase activity for each specimen. In practice, the values for the inhibited sections were low and relatively consistent. For example, the mean and the standard error for 20 different inhibited specimens were $13{\cdot}2 \pm 0{\cdot}7$, expressed as integrated OD units $\times 10^3$.

*Statistical evaluation of the measurements.*—The measurements from individual nerve sections of different animals with and without MISO (0·6 mg/g i.p., daily for 5 days) 4 weeks after commencement of treatment, were evaluated statistically. For the control animals, the variation between measurements on different animals could be accounted for by the variation in measurements on the individual sections. The treated animals, however, showed greater variation between animals than could be accounted for by variation within sections. It was therefore considered appropriate to express the results as means of groups of treated and untreated animals. Groups of 5 animals were used routinely for each treatment, and the standard error calculated for each. For example, a typical value for a group of control animals is $20 \pm 2$, and treated animals, $45 \pm 4$.

### RESULTS

### *β-Glucuronidase activity in different tissues after MISO treatment*

The β-glucuronidase activity was measured in various tissues of 30–50-week-old male C57 mice after MISO treatment (0·5 mg/g i.p., daily for 7 days). The areas of enzyme activity in the liver, kidney cortex, heart, cerebral cortex and untreated peripheral nerve, divided arbitarily into proximal and distal regions, were homogenous and selected at random for measurement. However, the areas of activity in the treated nerves were heterogeneous, and areas of maximum activity were selectively measured. On this basis no significant change in enzyme activity was seen in tissues other than the peripheral nerve, at 1 and 4 weeks after the start of treatment. However, increased activity was evident in the nerves at 4 weeks, the activity being greater in distal than proximal regions (Table I).

TABLE I.—*The β-glucuronidase activity in different tissues* 1 *and* 4 *weeks after commencement of MISO treatment* (0·5 *mg/g i.p., daily for* 7 *days*). *Values are expressed as means* ± *s.e.*

| Tissue | Controls (saline) | Week 1 | Week 4 |
|---|---|---|---|
| Liver | $84 \pm 6$ | $92 \pm 6$ | $71 \pm 6$ |
| Kidney | $50 \pm 4$ | $50 \pm 5$ | $48 \pm 4$ |
| Heart | $18 \pm 2$ | $18 \pm 1$ | $17 \pm 1$ |
| Cerebral cortex | $16 \pm 1$ | | $16 \pm 1$ |
| Proximal nerve | $26 \pm 2$ | $28 \pm 1$ | $37 \pm 4$ |
| Distal nerve | $26 \pm 2$ | $43 \pm 3$ | $57 \pm 2$ |

This latter observation is thought to explain the heterogeneous areas of activity seen in the nerves, which necessitated the selection of areas of activity, for the procedure of folding the nerve in order to embed it in kidney for sectioning results in areas of different distal location occurring in the same section. Alternative explanations suggested to us, are that different nerve fibres may vary in sensitivity or that there may be an influx of phagocytic mast cells.

In view of the above, further experiments to investigate neurotoxicity were performed only with the distal region of nerves. The β-glucuronidase activity was measured 4 weeks from the commencement of MISO dosing, since peak activity

FIG.—The effect of increased daily doses of 0·6 mg/g MISO on the β-glucuronidase activity in the peripheral nerves of mice 4 weeks after commencement of treatment.

has previously been shown to occur at this time (Clarke *et al.*, 1980).

*Effect of numbers of doses of MISO on the β-glucuronidase activity in distal peripheral nerves*

The Figure shows the effect of increasing the number of i.p. doses of MISO on the β-glucuronidase activity in 8–10-week-old female C57 mice. This activity was significantly elevated by only a single MISO dose of 0·6 mg/g. The activity increased with the number of daily doses, though the rate of increase fell. In view of this, in future work a regime of 5 daily doses was adopted, since it would give maximum activity with the minimum of drug.

*Variation of the route of administration of MISO*

The increase in β-glucuronidase activities at 4 weeks in the distal portions of nerves of 8–10-week-old C57 female mice treated with 0·6 mg/g MISO daily for 5 days, was similar whether the drug was administered i.p. (44 ± 3), orally (44 ± 1) or i.v. (43 ± 1). All values are given as integrated OD units $\times 10^3 \pm$ s.e.

*Effect of MISO on mice of different strains and sexes*

The β-glucuronidase activities were similar in the untreated male and female C57 mice, but the activities in untreated male WHT and CBA mice were greater than in females (Table II). In all cases, the enzyme activities measured 4 weeks after MISO treatment (0·6 mg/g i.p. daily for 5 days) were greater. The percentage increases ranged from: 126 (WHT ♀), 138 (WHT ♂), 142 (CBA ♂) and 167 (CBA ♀) to 203 (C57 ♂) and 221 (C57 ♀). Because of the greater increase in activity in the C57 ♀ mice after MISO treatment, these animals were used for most of the experiments in this study.

*Effect of methyl mercury and acrylamide*

The β-glucuronidase activity was increased in peripheral nerves 4 weeks after administration of 2 known neurotoxic agents, methyl mercury and acrylamide. Five daily doses of either methyl mercury ($7{\cdot}5 \times 10^{-3}$ mg/g/dose) or acrylamide ($50 \times 10^{-3}$ mg/g/dose) gave increased β-glucuronidase levels of 34 ± 4 and 32 ± 4 respectively, compared to the control value of 18 ± 2. These increases in enzyme activity were produced by 100-fold and 10-fold less drug, respectively, than that of MISO used above.

### DISCUSSION

The raised β-glucuronidase activities in

TABLE II.—*The effect of MISO on the β-glucuronidase activity in the peripheral nerves of different strains and sexes of mice 4 weeks after commencement of treatment* (0·6 *mg/g i.p., daily for* 5 *days*). *Values are expressed as means ± s.e.*

| Mouse strains | Untreated ♂ | MISO ♂ | Untreated ♀ | MISO ♀ |
|---|---|---|---|---|
| C57 | 23 ± 1·5 | 47 ± 5 | 20 ± 1 | 43·5 ± 3·5 |
| WHT | 33 ± 1 | 45 ± 5 | 26 ± 1·5 | 33 ± 3·5 |
| CBA | 36 ± 2 | 52 ± 8 | 21·5 ± 1·5 | 36·5 ± 2·5 |

murine nerves after MISO treatment, reported earlier (Clarke *et al.*, 1980) and again in this paper, appear to be specific to peripheral nervous tissue. They were not found in liver, kidney, heart or cerebral cortx, examined either at the peak time of 4 weeks after commencement of treatment or at 1 week. It is of interest that the $\beta$-glucuronidase activity varied between the different untreated tissues, with liver and kidney much more active than heart, cerebral cortex and peripheral nerves, especially when the shorter incubation times for the first 2 tissues are taken into consideration. These variations may well reflect the degree of metabolic activity of the tissue examined, with liver and kidney being more active than heart, cerebral cortex and peripheral nerves.

The time course of changes in lysosomal enzyme activity after MISO treatment was reflected in a behavioural study, measuring murine gait deficiency on a narrowing bridge (Clarke *et al.*, 1980). This led us to consider that the assay did provide a measure of neurotoxicity in the mouse. Further support is given to this argument from our observations, reported here, that 2 known neurotoxic agents, methyl mercury and acrylamide, also raised $\beta$-glucuronidase levels. Similar effects of these agents have been reported in rats (Dewar & Moffett, 1979). When it is considered that the administered doses of these agents are of 1–2 orders of magnitude less than our doses of MISO, this radiosensitizer can be considered relatively non-neurotoxic. Furthermore, preliminary data show that methyl mercury and acrylamide may produce a greater lysosomal response before the 4-week interval at which the effect of MISO is maximal. The above evidence has established this to be a reliable method of measuring MISO neurotoxicity, and we have carried out a series of experiments to optimize this assay. One such experiment has shown that the mouse strain and sex are important to the sensitivity of this assay, the $\beta$-glucuronidase activity being twice as great in untreated male CBA mice than female C57 mice, and the increase after MISO ranging from 26% in female WHT to 121% in female C57. For this reason, the latter animals were adopted for use in subsequent experiments.

This large variation in sensitivity between different mouse strains and sexes is not perhaps unexpected. For instance, acute $LD_{50}$ values for mice have been shown to differ by more than 70%, depending on mouse strain and in a given strain by more than 60% depending on body weight (Brown *et al.*, 1978, Denekamp, personal communication). Furthermore, Conroy *et al.* (1982) have reported that to achieve the same neurotoxic effect, BALB/Cba mice require twice the neural-tissue exposure of C3H mice. These differences have been attributed to the dependence of the pharmacology on mouse strain and sex. It is clear that comparisons between the relative neurotoxicity of different radiation sensitizers can only be made within the same sex and strain of mice.

The different routes of administration studied (oral, i.p. or i.v.) did not affect the increased $\beta$-glucuronidase activity from MISO treatment (0·6 mg/g/i.p., daily for 5 days). However, 10 similar daily doses of MISO did not significantly increase the $\beta$-glucuronidase activity above that after 5 doses, indicating that the effect had reached a plateau. Therefore, at lower doses of MISO than were used here differences between routes of administration might occur.

In conclusion, cytochemical measurement of increased $\beta$-glucuronidase activity in distal regions of the anterior and posterior tibial nerves has been shown to be a method suitable for measuring the neurotoxicity of MISO and, presumably, other potential hypoxic cell radiosensitizers. The system we have developed for this is to administer to female C57 mice 5 daily i.p. MISO doses and to measure the enzyme activity at 4 weeks after the commencement of dosing.

The authors wish to express their thanks to Professor E. D. Wills, Department of Chemistry and Biochemisty, the Medical College of St Bartholomew's Hospital, London, for use of the Scanning and Integrating Microdensitometer; Dr I. J. Stratford for constructive discussion; Dr N. M. Blackett for advice with the statistics; Mr J. Currant and Miss Dawn Scottow for excellent technical assistance; Mrs Annabel Thomas for typing; Professor G. E. Adams for his support and encouragement; Dr C. E. Smithen of Roche Products for the misonidazole, and the Medical Research Council and National Cancer Institute (Contract Number NOI-CM-77135) for financial support.

### REFERENCES

ADAMS, G. E. (1978) Hypoxic cell sensitizers for radiotherapy. *Int. J. Radiat. Oncol. Biol. Phys.*, **4**, 135.

ADAMS, G. E., DAWSON, K. B. & STRATFORD, I. J. (1980) Electron-affinic radiation sensitizers for hypoxic cells: Prospects and limitations with present and future drugs. *Proc. Int. Meeting for Radio-Cnɔology*, 1978, Vienna. Stuttgart: Georg Thieme. p. 84.

BITENSKY, L., BUTCHER, R. G. & CHAYEN, J. (1973) Quantitative cytochemistry in the study of lysosomal function. In *Lysosomes in Biology and Pathology*, Vol. 3, (Ed. Dingle). Amsterdam: North Holland. p. 465.

BROWN, J. M., YU, N. Y., CORY, M. J., BICKNELL, R. B. & TAYLOR, D. L. (1978) *In vivo* evaluation of radio-sensitizing and cytotoxic properties of newly synthesised electron-affinic drugs. *Br. J. Cancer*, **37**, 206.

CAVANAGH, J. B. (1973) Peripheral neuropathy caused by chemical agents. *CRC Crit. Rev. Toxicol.*, **2**, 365.

CHAYEN, J., BITENSKY, L. & BUTCHER, R. (1973) The preparation of sections. In *Practical Histochemistry*, Pt. 1. London: Wiley. p. 155.

CLARKE, C., DAWSON, K. B., SHELDON, P. W., CHAPLIN, D. J., STRATFORD, I. J. & ADAMS, G. E. (1980) A quantitative cytochemical method for assessing the neurotoxicity of misonidazole. In *Radiation Sensitizers: Their Use in the Clinical Management of Cancer* (Ed. Brady). New York: Masson. p. 245.

CONROY, P. J., VON BURG, T., PASSALACQUA, W., PENNY, D. P. & SUTHERLAND, R. M. (1979) Misonidazole neurotoxicity in the mouse: Evaluation of functional, pharmacokinetic, electrophysiologic and morphologic parameters. *Int. J. Radiat. Oncol. Biol. Phys.*, **5**, 983.

CONROY, P. J., MCNEILL, T. H., PASSALACQUA, W., MERRITT, J., REICH, K. R. & WALKER, S. (1982) Nitroimidazole neurotoxicity: Are mouse studies predictive? *Int. J. Radiat. Oncol. Biol. Phys.* (in press).

DEWAR, A. J. & MOFFETT, B. J. (1979) Biochemical methods for detecting neurotoxicity: A short review. In *Pharmacological Methods in Toxicology*, (Eds. Zbinden & Gross). Oxford: Pergamon. p. 545.

DISCHE, S., SAUNDERS, M. I., ANDERSON, P. & 6 others (1978) The neurotoxicity of misonidazole: Pooling of data from five centres. *Br. J. Radiol.*, **51**, 1023.

GRIFFIN, J. W., PRICE, D. L., KNETHE, O. D. & GOLDBERG, A. M. (1979) Neurotoxicity of misonidazole in rats. I. Neuropathy. *Neurotoxicology*, **1**, 299.

HIRST, D. G., VOJNOVIC, B., STRATFORDM I. J. & TRAVIS, E. L. (1978) The effect of the radiosensitizer misonidazole on motor nerve conduction velocity in the mouse. *Br. J. Cancer*, **37**, Suppl. III, 237.

HIRST, D. G., VOJNOVIC, B. & HOBSON, B. (1979) Changes in nerve conduction velocity in the mouse after acute and chronic administration of nitroimidazoles. *Br. J. Cancer*, **39**, 159.

ROSE, G. P. DEWAR, A. J. & STRATFORD, I. J. (1980) A biochemical method for assessing the neurotoxic effects of misonidazole in the rat. *Br. J. Cancer*, **42**, 890.

SAUNDERS, M. I., DISCHE, S., ANDERSON, P. & FLOCKHART, I. R. (1978) The neurotoxicity of misonidazole and its relationship to dose, half-life and concentration in serum. *Br. J. Cancer*, **37**, Suppl. III, 268.

VON BURG, R., CONROY, P. J. & PASSALACQUA, W. (1979) Peripheral electrophysiological parameters in mice treated with misonidazole. *Br. J. Cancer*, **40**, 134.